# Association analysis of the LTA4H gene polymorphisms and pulmonary tuberculosis in 9115 subjects

**DOI:** 10.1016/j.tube.2010.11.001

**Published:** 2011-01

**Authors:** James Curtis, Liliya Kopanitsa, Emma Stebbings, Arran Speirs, Olga Ignatyeva, Yanina Balabanova, Vladyslav Nikolayevskyy, Sven Hoffner, Rolf Horstmann, Francis Drobniewski, Sergey Nejentsev

**Affiliations:** a Department of Medicine, University of Cambridge, Cambridge, United Kingdom, Level 5, Addenbrookes Hospital, Hills road, Cambridge CB2 0QQ, United Kingdom; b Samara Oblast Tuberculosis Dispensary, Samara, Russian Federation, Russia; c Health Protection Agency National Mycobacterium Reference Laboratory and Clinical TB and HIV Group, Institute for Cell and Molecular Sciences, Barts and the London School of Medicine, Queen Mary, University of London, London, UK; d Department of Bacteriology, Swedish Institute for Infectious Disease Control, Solna, Sweden; e Department of Molecular Medicine, Bernhard Nocht Institute for Topical Medicine, 20359 Hamburg, Germany

**Keywords:** Tuberculosis, Gene, SNP, Association, Heterozygosity

## Abstract

Immunoregulatory eicosanoids have been implicated in protection from mycobacterial infection in cell and animal models. Recently, a study of the zebrafish embryo demonstrated that mutants of the *lta4h* gene, which encodes the leukotriene A4 hydrolase (LTA4H) enzyme of the eicosanoid pathway, have hypersusceptibility to Mycobacterium marinum infection. It also reported that heterozygosity at the two single nucleotide polymorphisms rs1978331 and rs2660898 located in introns of the LTA4H gene, a human homologue of *lta4h*, is associated with protection from pulmonary tuberculosis. To replicate this association we genotyped six LTA4H gene polymorphisms in samples from 3703 pulmonary tuberculosis patients and 5412 healthy controls collected in Russia. We found no evidence of the protective effect of heterozygosity at the polymorphisms rs1978331 and rs2660898 (*P* = 0.29 and 0.49) and no association of the alleles of any of the six polymorphisms (*P* = 0.13–0.81). These results suggest that common polymorphisms in the LTA4H gene do not play any major role in susceptibility to clinical pulmonary tuberculosis.

## 1 Introduction

With approximately 9 million new cases and more than 1 million deaths tuberculosis (TB) remains a global health problem.^1^ Although one third of the world’s population is estimated to be infected with *Mycobacterium tuberculosis*, less than 10% of the immuno-competent individuals progress to clinical disease. Host susceptibility is, therefore, considered to be an important risk factor for the development of active TB after *M. tuberculosis* infection, yet its underlying biological mechanisms remain largely unknown. Several lines of evidence indicate that genetic factors play an important role in susceptibility to TB. For example, inbred animal strains demonstrate variable degree of susceptibility or resistance to *M. tuberculosis* infection.^2^ In humans monozygotic twins have higher TB concordance than dizygotic twins.^3,4^ Furthermore, studies in human patients that have susceptibility to the weakly virulent mycobacteria discovered rare mutations in several immune genes, collectively known as Mendelian susceptibility to mycobacterial disease (MSMD) syndrome.^5^

Given the difficulties in studying the molecular biology of TB in humans, much of our knowledge about mycobacterial infection originates from animal models, especially mouse strains and guinea pigs infected with *M. tuberculosis*^2^ and, more recently, zebrafish infected with its natural pathogen *Mycobacterium marinum,* a close relative of *M. tuberculosis*.^6^ In a recent study Tobin et al. performed a forward genetic screen in zebrafish embryo and discovered a class of mutants hypersusceptible to *M. marinum* infection, which displayed increased bacterial growth and reduced TNF signalling and apparently was caused by mutations in the *lta4h* gene.^7^ This gene encodes leukotriene A4 hydrolase (LTA4H) that is involved in the synthesis of leukotriene B_4_ (LTB_4_), a proinflammatory eicosanoid and a powerful chemoattractant. Mutations of the *lta4h* gene reduced its expression and conferred hypersusceptibility to *M. marinum* by redirecting leukotriene A4 (LTA_4_), the LTB_4_ precursor, to production of the anti-inflammatory lipoxin A4 (LXA_4_).^7^ These data are in line with previous findings that implicated immunoregulatory eicosanoids in protection from mycobacterial infection in cell and animal models. Thus, LXA_4_ is known to reduce resistance of mice to *M. tuberculosis* infection.^8^ Virulent *M. tuberculosis* have been shown to increase LXA_4_ production, which downregulated prostaglandin E_2_ that functions to activate membrane repair and protect macrophages from *M. tuberculosis*-induced necrosis.^9,10^

Tobin et al. then undertook a human genetic study, in which they investigated polymorphisms in the LTA4H gene, a human homologue of *lta4h*, for association with clinical TB and leprosy phenotypes. They reported that heterozygosity at two intronic single nucleotide polymorphisms (SNPs) in the LTA4H gene is associated with protection from pulmonary and meningeal TB and multibacillary leprosy without erythema nodosum leprosum.^7^ In genetic association studies independent replication in large sample collections is essential, given a large number of false associations between sequence polymorphisms and various complex diseases that have been reported previously.^11,12^ Therefore, here we studied a collection of 9115 samples from pulmonary TB patients and healthy controls in order to confirm or refute association between LTA4H polymorphisms and pulmonary TB.

## 2 Materials and methods

Pulmonary TB patients attending civilian TB dispensaries and TB clinics along with healthy subjects attending the blood transfusion service were recruited in two Russian cities, St. Petersburg and Samara, as described previously.^13^ TB patients have been diagnosed using information about TB contact, medical history and clinical symptoms (cough, haemoptysis, chest pain, fever, weight loss), presence of acid fast bacilli in sputum smear and had symptoms characteristic of pulmonary TB on chest X rays. At the time of diagnosis cavities were present in 70% of the patients. For all patients in this study diagnosis has been confirmed by culture of *M. tuberculosis* from sputum. We excluded from the study patients with extra-pulmonary TB and all HIV-positive subjects. Controls were healthy adult blood bank donors with no history of TB. In total we studied 3703 culture-confirmed pulmonary TB patients and 5412 controls, including 2004 patients and 2792 controls from St. Petersburg and 1699 patients and 2620 controls from Samara.

We extracted genomic DNA from whole blood of the participating subjects using a standard chloroform/proteinase K protocol. We checked DNA quality using 1% agarose gel electrophoresis, determined DNA concentration using Picogreen reagent and then normalised concentration for genotyping. We genotyped six LTA4H SNPs using custom Taqman assays and 7900HT system from Applied Biosystems (Table S1). We visually checked all genotype clusters and assigned calls using SDS 2.3 software.

Allelic association analysis was done using Stata version 11. Odds ratios and *P*-values were calculated by Mantel–Haenszel test controlling for the city of the sample origin (St. Petersburg or Samara). Statistical power calculations were done using EPITABLE in Epi-info version 6 and Genetic Power Calculator.^14^

## 3 Results

We genotyped six LTA4H gene SNPs in 3703 HIV-negative adult pulmonary TB patients and 5412 healthy controls from Russia. Genotypes did not deviate from Hardy–Weinberg equilibrium beyond that expected at random (Table 1). Initially we studied the allelic effects of all six LTA4H SNPs, but found no evidence of association with TB for any of them (Table 1). We then compared frequencies of heterozygotes versus homozygotes among TB cases and controls for the rs1978331 and rs2660898 SNPs in order to confirm or refute protective effects of heterozygotes reported by Tobin et al. We found no evidence of association: for rs1978331 odds ratio (OR) = 0.98, 95% confidence interval (95% CI) 0.90–1.06, *P*_one-tailed_ = 0.29; for rs2660898 OR = 1.0, 95% CI = 0.91–1.09, *P*_one-tailed_ = 0.49 (genotype counts are shown in Table 1). We did not test heterozygosity of the other four SNPs for association with TB because they were not associated with TB previously^7^ and because a biological model where heterozygotes confer protection, while both homozygotes confer susceptibility, is unlikely. A statistical test comparing numbers of subjects heterozygous at both rs1978331 and rs2660898 and those that are homozygous in at least one SNP also did not reveal any significant difference between TB patients and controls (proportions of double heterozygotes were 38.7% and 39.1%, respectively; *P* = 0.75).

## 4 Discussion

Tobin et al. genotyped six SNPs of the LTA4H gene in 658 pulmonary and meningeal TB patients and 751 controls from Vietnam and reported that protection from both pulmonary and meningeal TB is associated with heterozygosity at two intronic SNPs, rs1978331 and rs2660898.^7^ Here we found no association between the same six SNPs and pulmonary TB in the Russian population. Our study had 100% statistical power to detect at *P*_one-tailed_ = 0.05 protective effects of heterozygotes with OR = 0.64 and 0.71 reported for the rs1978331 and rs2660898 SNPs, respectively.^7^ Our study was also well powered to detect weaker allelic effects, e.g. > 90% power for OR ≤ 0.85 for alleles of all six SNPs at *P*_two−tailed_ = 0.05, assuming a multiplicative genetic model. Therefore, it is very unlikely that we could miss a genuine association in the Russian population. Of course, our study does not replicate every aspect of the genetic association experiment done by Tobin et al. In particular, here we studied a Russian population that has a European origin and is distinct from the Vietnamese and Nepali populations, where associations with TB and leprosy, respectively, have been reported.^7^ Although differential effects of genetic polymorphisms on susceptibility to disease in various populations cannot be excluded, we note that the choice of SNPs by Tobin et al. was driven by the association of the LTA4H SNPs with the level of LTB4 production that was originally reported in Icelanders, another European population.^15^ This suggests that, if LTA4H SNPs are indeed functional and influence susceptibility to TB, their effects would be likely to be seen across different populations. We also note that statistical evidence of association between LTA4H SNPs and several TB and leprosy phenotypes reported by Tobin et al. is rather weak (*P* = 0.021–4 × 10^−5^). Such associations are not uncommon when multiple polymorphisms across many candidate genes are tested. However, historically, genotype-phenotype associations that do not reach strong statistical evidence (e.g. *P* < 10^−7^) have rarely been replicated, suggesting that many of them arise due to chance, a phenomenon that has been attributed to a low prior probability of genetic association in complex diseases, multiple statistical testing and relatively small datasets in the initial association reports.^16–19^ Inconsistent associations have been a problem in TB genetics, e.g. associations in the SP110 and MAL/TIRAP genes have been reported,^20,21^ but were not confirmed in the subsequent studies.^13,22–24^ A lack of replication in this large sample collection indicates that common LTA4H polymorphisms have no major effect on pulmonary TB, at least in the Russian population. Further studies in statistically powerful sample collections from Asia are required to definitively conclude on the role of LTA4H in susceptibility to TB. Tobin et al. also reported an even weaker statistical evidence suggesting that heterozygosity at the LTA4H SNPs is associated with lower mortality among patients with meningeal TB, as well as protection from developing a multibacillary leprosy without erythema nodosum leprosum (*P* = 0.025 and 0.001, respectively). Here we did not address these questions and they remain to be formally tested.

Studies of animal models, including zebrafish, have been extremely informative in uncovering underlying mechanisms of mycobacterial infection.^25,26^ However, because of the natural differences between these models and humans, relevance of particular biological pathways to clinical TB is often difficult to demonstrate. Human genetic studies can address this crucial question at the aetiological level. Thus, discovery of a genuine association between a genetic variant and a disease indicates that a causative genetic factor exists in the vicinity of the associated variant and, when mapped to a particular gene, proves its aetiological contribution to human disease. Nevertheless, statistically convincing and replicable evidence of association is a prerequisite. Therefore, it still remains to be shown whether genetic variation in LTA4H or other genes of the eicosanoid pathway plays any role in susceptibility to clinical TB.

## Funding

SN is a Royal Society University Research Fellow. This study has been supported by the Royal Society Research grant, the Wellcome Trust grant WT088838MA and the European Union Framework Programme 7 grant 201483 to the TB-EUROGEN consortium (FD, SN, RH and SH).

## Competing interests

None declared.

## Ethical approval

This work has been approved by Human Biology Research Ethics Committees of the University of Cambridge and of Queen Mary College, London and the local Ethics Committees in St. Petersburg and Samara, Russia. All participants provided a written informed consent before being enrolled into the study.

## Figures and Tables

**Table S1:** Probes and primers of the Taqman assays used for genotyping LTA4H SNPs

SNP		Sequence
rs1978331	Forward Primer	TGGAAAGTGAAGTCAATGCTGGTAT
	Reverse Primer	GCTCCCTATTTAGACCAGGTCATCA
	Reporter (VIC)	CATAGAACAGTGAAC**G**GTGTG
	Reporter (FAM)	ATAGAACAGTGAAC**A**GTGTG
rs17677715	Forward Primer	AACAAAAAATTAAGAAGGAAGGGCACTTT
	Reverse Primer	ACTTGGAAGCCTACAAATATTACAGAAATGT
	Reporter (VIC)	AAGATATTTTATCA**G**TATTTTAC
	Reporter (FAM)	AAGATATTTTATCA**A**TATTTTAC
rs2660898	Forward Primer	TCATAAATTTGGAGGATCAAACTTCAGTGT
	Reverse Primer	GCTGAAGGAGACTTAATCGGACAAA
	Reporter (VIC)	CCCAAAACT**G**GAAAAG
	Reporter (FAM)	AACCCAAAACT**T**GAAAAG
rs2247570	Forward Primer	GTGCAGCAATCTTGGGATAAGTG
	Reverse Primer	GTGTAGATTATACCAGCAGGGTTACTG
	Reporter (VIC)	AAATGCATTCTTGT**A**TCTAAT
	Reporter (FAM)	TGCATTCTTGT**G**TCTAAT
rs2660845	Forward Primer	ACTTCCTGTGGACTTCATAGTGTCT
	Reverse Primer	GGTGTATCGAGCCACTCAAGT
	Reporter (VIC)	CCACTGGCCCC**A**CGGG
	Reporter (FAM)	CACTGGCCCC**G**CGGG
rs2540475	Forward Primer	GCCAGCAGTTGTAAAGAACTTTTTCT
	Reverse Primer	CCTTCTCCCTACCCCAGCTT
	Reporter (VIC)	CCCTCCCTA**C**TTCATC
	Reporter (FAM)	CCCTCCCTA**T**TTCATC

**Table 1:** LTA4H gene SNPs in Russian pulmonary TB patients and controls.

Genotype counts (frequencies, %)												
SNP		n	0.0		0.1		1.1		*P*_HWE_	MAF (%)	OR∗ (95% CI)	*P*-value†
rs1978331	TB	3601	1267	(35.2)	1729	(48.0)	605	(16.8)	0.71	40.8	1.05 (0.99–1.11)	0.13
*A* = 0 *G* = 1	Controls	5238	1887	(36.0)	2546	(48.6)	805	(15.4)	0.26	39.7		
rs17677715	TB	3599	2593	(72.0)	927	(25.8)	79	(2.2)	0.72	15.1	1.06 (0.98–1.16)	0.15
*A* = 0 *G* = 1	Controls	5247	3851	(73.4)	1292	(24.6)	104	(2.0)	0.72	14.3		
rs2247570	TB	3595	1859	(51.7)	1437	(40.0)	299	(8.3)	0.37	28.3	1.03 (0.97–1.10)	0.34
*A* = 0 *G* = 1	Controls	5268	2751	(52.2)	2123	(40.3)	394	(7.5)	0.57	27.6		
rs2660898	TB	3604	1607	(44.6)	1590	(44.1)	407	(11.3)	0.65	33.4	1.05 (0.98–1.12)	0.18
*T* = 0 *G* = 1	Controls	4894	2230	(45.6)	2160	(44.1)	504	(10.3)	0.57	32.4		
rs2660845	TB	3595	258	(7.2)	1400	(38.9)	1937	(53.9)	0.82	26.6	1.01 (0.94–1.08)	0.81
*G* = 0 *A* = 1	Controls	5264	371	(7.0)	2050	(38.9)	2843	(54.0)	0.96	26.5		
rs2540475	TB	3517	2385	(67.8)	1009	(28.7)	123	(3.5)	0.20	17.8	0.98 (0.91–1.06)	0.66
*C* = 0 *T* = 1	Controls	5198	3458	(66.5)	1597	(30.7)	143	(2.8)	0.01	18.1		

SNP – single nucleotide polymorphism, MAF – minor allele frequency, OR – odds ratio, 95% CI – 95% confidence interval.

∗ – OR and 95% CI for minor alleles are shown.

† – *P*-values for the allelic association were calculated by Mantel–Haenszel test controlling for the origin of the sample.
